# DFMO inhibition of neuroblastoma tumorigenesis

**DOI:** 10.1002/cam4.7207

**Published:** 2024-04-30

**Authors:** Divya Gandra, David H. Mulama, David M. Foureau, Kimberly Q. McKinney, Elizabeth Kim, Kaitlyn Smith, Jason Haw, Abhinav Nagulapally, Giselle L. Saulnier Sholler

**Affiliations:** ^1^ Department of Pediatrics Penn State Health Children's Hospital Hershey Pennsylvania USA; ^2^ Department of Pediatrics Levine Children's Hospital Charlotte North Carolina USA; ^3^ Department of Medicine Levine Cancer Institute Charlotte North Carolina USA

**Keywords:** cell cycle, DFMO, ELDA, neuroblastoma, neurosphere, senescence, xenograft

## Abstract

**Background:**

Most high‐risk neuroblastoma patients who relapse succumb to disease despite the existing therapy. We recently reported increased event‐free and overall survival in neuroblastoma patients receiving difluoromethylornithine (DFMO) during maintenance therapy. The effect of DFMO on cellular processes associated with neuroblastoma tumorigenesis needs further elucidation. Previous studies have shown cytotoxicity with IC50 values >5–15 mM, these doses are physiologically unattainable in patients, prompting further mechanistic studies at therapeutic doses.

**Methods:**

We characterized the effect of DFMO on cell viability, cell cycle, apoptosis, neurosphere formation, and protein expression in vitro using five established neuroblastoma cell lines (BE2C, CHLA‐90, SHSY5Y, SMS‐KCNR, and NGP) at clinically relevant doses of 0, 50, 100, 500, 1000, and 2500 μM. Limiting Dilution studies of tumor formation in murine models were performed. Statistical analysis was done using GraphPad and the level of significance set at *p* = 0.05.

**Results:**

There was not a significant loss of cell viability or gain of apoptotic activity in the in vitro assays (*p* > 0.05). DFMO treatment initiated G1 to S phase cell cycle arrest. There was a dose‐dependent decrease in frequency and size of neurospheres and a dose‐dependent increase in beta‐galactosidase activity in all cell lines. Tumor formation was decreased in xenografts both with DFMO‐pretreated cells and in mice treated with DFMO.

**Conclusion:**

DFMO treatment is cytostatic at physiologically relevant doses and inhibits tumor initiation and progression in mice. This study suggests that DFMO, inhibits neuroblastoma by targeting cellular processes integral to neuroblastoma tumorigenesis at clinically relevant doses.

## 1 BACKGROUND

Neuroblastoma (NB) is the most common extracranial tumor, accounting for 8%–10% of pediatric cancers, and 15% of cancer‐related deaths in children in the United States.^1^, ^2^ Over 700 NB cases are reported in the United States annually, with a median age at diagnosis of 18 months.^3^ Current treatment approaches for high‐risk neuroblastoma (HRNB) include surgery, chemotherapy, radiation, bone marrow transplant, and immunotherapy.^4^ Despite this multimodal treatment, HRNB prognosis is poor with 50% relapse rate in high‐risk patients, of whom over 90% succumb to disease. This malignancy originates from undifferentiated neural crest cells of the sympathetic nervous system.^5^ The degree of risk is associated with age of the patient, disease stage, histopathologic results, and tumor genomics.^4^

The most common and well‐characterized biomarker for HRNB is the *MYCN* oncogene amplification.^6^, ^7^ Patients with *MYCN* gene amplification, who account for 30% of those diagnosed with NB, report poor prognosis.^8^ The *MYCN* gene impacts several cellular processes integral to tumorigenesis.^8^, ^9^ *MYCN* regulates polyamine metabolism via the LIN28B‐Let7‐*MYC*N axis. The inhibition of polyamine biosynthesis represses LIN28B‐Let7‐*MYCN* signaling pathway and abrogates cell proliferation.^9^, ^10^ We hypothesize that the reduction in *MYCN* expression could be the mechanism for disease amelioration.

Ornithine decarboxylase‐1 (ODC‐1), the rate limiting enzyme in polyamine biosynthesis, is overexpressed in NB.^11^ Polyamines are enzymatic by‐products of amino acids ornithine and arginine metabolism which are essential for cell proliferation and viability.^12^ Eflornithine (alpha‐difluoromethylornithine; DFMO) specifically and irreversibly inhibits the ODC‐1 pathway.^13^, ^14^ DFMO, initially developed as a trypanocide against African trypanosomiasis,^15^, ^16^ is currently under clinical trials as a repurposed maintenance therapy in many solid tumors including NB. DFMO treatment suppresses *MYCN* and LIN28 pathway resulting in an increase in Let7.^17^ Our group, through a Phase II clinical trial evaluating DFMO as maintenance therapy in HRNB patients, showed improved event‐free survival (EFS), and overall survival (OS) in subjects treated, after standard HRNB therapy, with 2 years of DFMO maintenance therapy.^18^ The 5‐year EFS and OS were 85.2% and 95.1% for the DFMO group subset versus 65.6% and 81.6% for the historical control group, respectively. This observation suggests that there may be a clinical benefit for DFMO as a maintenance therapy and provides rationale for further studies to understand the mechanism of action. Recent publications have shown that antineoplastics such as metformin can reduce proliferation and tumorigenic properties in NB by targeting tumor initiation process in vitro.^19^, ^20^ We hypothesized that DFMO similarly exerts its effects by targeting cellular processes integral to NB tumorigenesis. In this study, we show that DFMO induces cell cycle arrest and senescence and abrogates neurosphere formation in vitro. We recapitulate these findings in vivo using murine xenograft models which demonstrate the ability of DFMO to inhibit tumor formation and extend overall survival.

## 2 METHODS

### 2.1 Cell culture

Drug treatments were conducted in vitro using five established human NB cell lines: BE2C (CRL‐2268, purchased from ATCC, Manassas, VA), SMS‐KCNR (from the Children's Hospital of Philadelphia, PA), CHLA‐90 (from the Children's Hospital of Los Angeles, CA), SHSY5Y (ATCC CRL‐2266, NY), NGP (from Dr. Shohet's laboratory, MA). All cell lines were authenticated by short tandem repeat (STR) analysis (IDEXX Laboratories Inc., Westbrook, MO) and tested for mycoplasma before use. They were cultured in RPMI‐1640 supplemented with 10% (vol/vol) fetal bovine serum, 100 U/mL penicillin, and 100 mg/mL streptomycin, at 37°C with 5% CO_2_. All cell culture reagents were purchased from Gibco (Thermo Fisher Scientific, Waltham, MA).

### 2.2 DFMO drug reconstitution

DFMO (a kind gift from Dr. Patrick Woster's lab at the University of South Carolina, and CoreRX, Clearwater, FL) was reconstituted to 1M solution in sterile water, filtered, and stored in small aliquots at −20°C for subsequent dilution in culture medium for functional tests on NB cell lines in vitro. Escalating DFMO doses of 0, 50, 100, 500, 1000, and 2500 μM were used for all assays which were performed in triplicate and replicated at least three times. In vivo administration of DFMO for mouse studies was achieved by dissolving DFMO to 2% (w/v) in drinking water.

### 2.3 Flow cytometric analysis of cell viability, apoptosis and cell cycle

To investigate the effect of DFMO on cell cycle, cell viability, and apoptosis, 5 × 10^5^ cells per well were seeded in 6‐well plates for 2 days to adhere and acclimatize to culture conditions before treatment. After 48 and 72 h post‐treatment, the cells were harvested using TrypLE® Express (Thermo Fisher Scientific, Boston, MA), cellular matrix dissociated using 1 AccuMax (Innovated Cell Technologies, San Diego, CA) for 10 min, filtered through a 40 μm cell strainer (Thermo Fisher Scientific, Boston, MA) and then stained for apoptosis, cell viability and cell cycle. Vybrant™ FAM Caspase‐3 and‐7 Assay kit (Invitrogen, Eugene, OR #V35118) and Dye Cycle Violet (Invitrogen, Eugene, OR #C10094) was used to stain for apoptosis and cell cycle analysis following the manufacturer's recommendations. Cell viability was investigated by dye exclusion principle using Live/Dead Fixable Far red fluorescent reactive dye (Invitrogen, Boston, MA #L34973). Flow cytometry was conducted and acquired on BD LSR Fortessa (BD Biosciences, Franklin Lakes, NJ), and the FACS data analyzed using FlowJo ver10.9(BD Biosciences, Franklin Lakes, NJ). We performed cytotoxicity assay and cell cycle analysis at both 48 and 72 h post‐treatment. For each cell line/time point, we compiled data from independent experiments and tested each DFMO concentration in duplicate or triplicate each time (*n* = 4–5 per datapoint). Descriptive data for the viability and apoptosis assays were generated using FlowJo. Cell cycle analysis was analyzed in FlowJo using Watson pragmatic algorithm.

### 2.4 The effect of DFMO on senescence‐associated ß‐galactosidase activity in NB

To investigate the effect of DFMO on cell senescence, 10,000 cells per well were seeded in 6‐well plates and allowed to adhere overnight. The cells were treated with escalating doses of DFMO as indicated above, or with 12.5 μM Etoposide as a positive control, for 48 and 72 h. Senescence‐associated ß‐galactosidase (SA‐βGal) activity was performed using a commercially available kit (Cell Signaling Technology, Danvers, MA #9860). Cells were washed, fixed, and exposed to staining solution at pH 6.0 for 16 h at 37°C. The solution was then replaced with 70% glycerol for long‐term storage. Five field images per well were then captured at 20× magnification on a Nikon Eclipse TS100 microscope affixed with a Nikon DS‐Fi2 color camera. The number of SA‐βGal positive (blue‐stained) cells were manually counted against the total number of cells in each field of view to calculate mean percent positive cells.

### 2.5 The effect of DFMO on neurosphere formation in NB

To investigate the effect of DFMO on tumor initiation potential, NB cells either from established cell lines or excised from xenograft tumors were used for neurosphere assay. For the in vitro experiments, cells (derived from the five established cell lines or from mouse tumors) were seeded at a density of 2 cells per well in 96‐well ultra‐low attachment round bottom plates (Corning Life Sciences, Corning, NY) in RPMI 1640 culture medium supplemented with 10% (vol/vol) fetal bovine serum, 100 U/mL penicillin, and 100 mg/mL streptomycin. Increasing concentrations of DFMO were added the following day and replenished every 3 days. The cells were imaged using Incucyte ZOOM® and monitored for 4 weeks. Both the frequency and size of neurospheres were recorded. The frequency of neurosphere formation was calculated by dividing the number of wells with neurospheres by the total number of wells plated. Neurosphere size was determined by calculating the area of the neurosphere using the following generalized ellipsoid formula (*π* * *A* * *B*) where *A* is the major radius and *B* is the minor radius.

### 2.6 The effect of DFMO treatment on cell cycle and tumor‐associated protein expression

To investigate the effect of DFMO on the expression of proteins involved in cell cycle, we performed western blot analysis on protein lysates created from DFMO‐treated cultured cells or protein lysates made from excised mouse tumors. Tumor lysates were created by mechanical homogenization of tumor in RIPA buffer. The resulting slurry was then incubated on ice for 30 min, followed by centrifugation at 16,000× *g* for 15 min. Supernatants were transferred to clean tubes and spins repeated until a clear lysate was achieved.

For in vitro experiments, cells were seeded at a density of 1 × 10^5^ cells per well in 6‐well plates. DFMO treatment commenced when cultures attained 40%–50% confluency. The DFMO concentrations used were 0, 50, 100, 500, 1000, and 2500 μM. After 48 and 72 h of DFMO treatment, the cells were harvested and lysed in RIPA buffer containing protease and phosphatase inhibitors (Thermo Fisher Scientific, Boston, MA) before short‐term storage at −80°C. The cell pellets were then probe sonicated, incubated for 30 min on ice, and centrifuged to clear the lysates. Protein concentrations were determined by Bicinchoninic acid (BCA) assay. Proteins were resolved by electrophoresis on 4%–20% Bis‐Tris polyacrylamide gels, then dry transferred onto a nitrocellulose membrane using the iBLOT (Thermo Fisher Scientific, Boston, MA). After blocking, membranes were incubated in primary antibodies directed against cell cycle‐associated proteins Cyclin D1 (EMD Millipore, Burlington, MA #ABE52), phospho‐Rb (#9308) and Total Rb (#9309) as well as β‐Actin (#4970) (Cell Signaling, Danvers, MA). Blots were washed and then incubated with goat anti‐rabbit (#7074) or goat anti‐mouse horseradish peroxidase‐conjugated secondary antibody (#91196) (Cell Signaling, Danvers, MA). Protein bands were detected by Radiance Plus chemiluminescence (Azure Biosystems, Dublin, CA) and imaged with the Azure 600 imaging system. Densitometry of the protein bands was performed using AzureSpot Software (Azure Biosystems, Dublin, CA) and expression of cell cycle proteins was normalized to ß‐actin expression. Fold change was calculated for each experimental replicate by comparing expression in treated cells to the vehicle control (0 μM DFMO). Mean fold change was then calculated.

For mouse tumor protein lysates, primary antibodies directed against NB‐associated proteins include rabbit monoclonal antibody to ODC1 (# ab126590), (Abcam, Cambridge, UK), rabbit monoclonal antibody to LIN28B (#4196), N‐MYC (#9405), and β‐Actin (#4970), (Cell Signaling, Danvers, MA). Goat anti‐rabbit and goat anti‐mouse horseradish peroxidase‐conjugated secondary antibody were used (Santa Cruz Biotechnology, Dallas, TX). Protein bands were detected by chemiluminescence with the ChemiDoc™ MP System (Bio‐Rad, Hercules, CA). Quantification of the protein bands was performed by comparing the relative expression of the protein band of interest to Actin using Image Lab™ Software, Version 5.2 (Bio‐Rad, Hercules, CA).

### 2.7 Animal care and husbandry

In vivo DFMO treatment studies were conducted in 4‐week‐old female nude mice (nu/nu) from Charles River Laboratories (Portage, MI). Mice were housed in pathogen‐free conditions and cared for in accordance with the institutional animal care and use protocol form (IACUC) approved by the West Michigan Regional Laboratory (WMRL). Mice were housed in Innovive IVC Innocages (San Diego, CA). Cages were pre‐bedded with corncob bedding and Innorichment sheet to enhance natural foraging behavior. Food and water were offered ad libitum while water was supplied using Innovive's Aquavive water bottles while food was irradiated Harlan Tekla diet (Indianapolis, IN). The mice were monitored daily for wellness and any signs of distress. Weights were assessed once weekly, while tumor volume was measured twice a week. Any mice exhibiting signs of physical deterioration or decline, such as >15% weight loss, dehydration, hunched posture, or max tumor burden were removed from the study and humanely euthanized according to the American Veterinary Medical Association approved euthanasia method of CO_2_ asphyxiation followed by cervical dislocation. Three xenograft models using BE2C and SMS‐KCNR were used as described (see experimental design schema in Figure S1).

### 2.8 Mouse xenograft experiments

#### 2.8.1 Limiting dilution assay xenograft (in vitro DFMO pretreated cells)

Female NSG mice (*n* = 180) were randomly assigned into two arms of study. Prior to subcutaneous injection, NB cells (BE2C and SMS‐KCNR) were incubated in culture medium at a concentration of 5000 μM DFMO for 20 days, 10 days, and 0 days. DFMO pretreatment was staggered such that all cells completed treatment on the day of injection into animals. Cells were harvested and resuspended in Matrigel (BD Biosciences, San Jose, CA). BE2C cells were injected in limiting dilutions of 10, 50, or 100 cells in a total volume of 100 μL Matrigel (10 mice in each group). SMS‐KCNR cells were injected in limiting dilutions of 500, 1000, or 5000 cells in a total volume of 100 μL Matrigel (10 mice in each group). Tumor formation in mice was monitored twice per week, measuring tumor volume by caliper method and calculated by the ellipsoid formula (length × width × height × 0.52 mm^3^).^21^ Masses were considered tumors once their volume had reached 100 mm^3^. The study ended after 60 days post‐injection for BE2C xenografts, and 90 days for SMS‐KCNR xenografts.

#### 2.8.2 Limiting dilution assay xenograft (in vivo DFMO treatment)

A total of 120 mice were used for the in vivo limiting dilution xenograft assay. The animals were randomly assigned into two equal groups of 60 mice each. The mice were injected with limiting dilutions of BE2C or SMS‐KCNR cells. BE2C cells (10, 50, and 100 cells) were injected into 30 mice receiving no treatment (*n* = 10 per cell dilution) and 30 mice receiving 2% DFMO (by volume) in drinking water (*n* = 10 per cell dilution). Similarly, SMS‐KCNR cells (500, 1000, and 5000 cells) were injected into 30 mice receiving no treatment and 30 mice receiving 2% DFMO in drinking water. The mice were given either regular drinking water or DFMO‐drinking water on the day of injection and thereafter until the end of the study, or 52 days for BE2C xenografts and 75 days for SMS‐KCNR xenografts. As above, tumor formation was monitored until the end of the study and tumor volume was measured twice a week using the caliper method, until euthanization.

### 2.9 In vivo DFMO treatment to assess progression of tumor in BE2C xenograft mice

Mice were injected with 2 × 10^6^ BE2C cells subcutaneously in their right flank. Once tumors were palpable (7 days post‐injection), mice were grouped into two treatment groups (*n* = 9) with equal average tumor sizes: untreated versus DFMO‐treated. The untreated group had access to normal drinking water, while the DFMO‐treated group received a solution of normal drinking water mixed with 2% DFMO (by volume) and changed every 3 days. After 7 days of treatment (14 days after injection) all mice were sacrificed to yield tumors for analysis for size, tumor volume, tumor imaging and histology. Neurosphere assay, and western blot was performed using cells from dissociated tumor cells.

### 2.10 Immunohistochemistry

To investigate the in vivo effect of DFMO treatment on expression of cell markers associated with NB tumorigenicity, mice were injected with 2 × 10^6^ BE2C cells and allowed to form tumors for 7 days, at which point mice received either untreated water or water supplemented with 2% DFMO for 7 days. After 7 days tumors were resected, and tumor volume measured. Tumors (*n* = 9) were excised and placed in 10% neutral‐buffered formalin for 24 h. After exposure to formalin, samples were paraffin imbedded and 5 μm slices of tissue made. All samples were stained for hematoxylin and eosin (tumor structure), Ki67 (cell division), p16ink4a (cellular senescence), LIN28B (marker of oncogenesis) and cleaved caspase 3 (cell death). All samples were processed, sliced, and stained by the Spectrum Health System IHC Core. Histology scoring was performed by a board‐certified histologist within Spectrum Health System IHC Core.

### 2.11 Statistical analysis

GraphPad Prism 9 (San Diego, USA) was used for all statistical analysis. Data for both in vitro and in vivo assays including flow cytometry analysis immunohistochemistry, SA‐βGal, are presented. For immunoblot band intensities (densitometry) were normalized to β‐actin as a loading control. All blots were then normalized to the average vehicle control before comparison. The likelihood for tumor formation in mouse experiments was calculated using Fisher exact test. All assays were conducted in triplicates and repeated at least three times. Data were compared using either two tailed Mann–Whitney *U*‐test and Kruskal–Wallis test followed by Dunn's post hoc test analysis to determine which groups were significantly different than the control groups with the level of significance set at *p* = 0.05.

## 3 RESULTS

### 3.1 DFMO treatment effect on cytotoxicity, apoptosis, and cell cycle arrest

**FIGURE 1:**
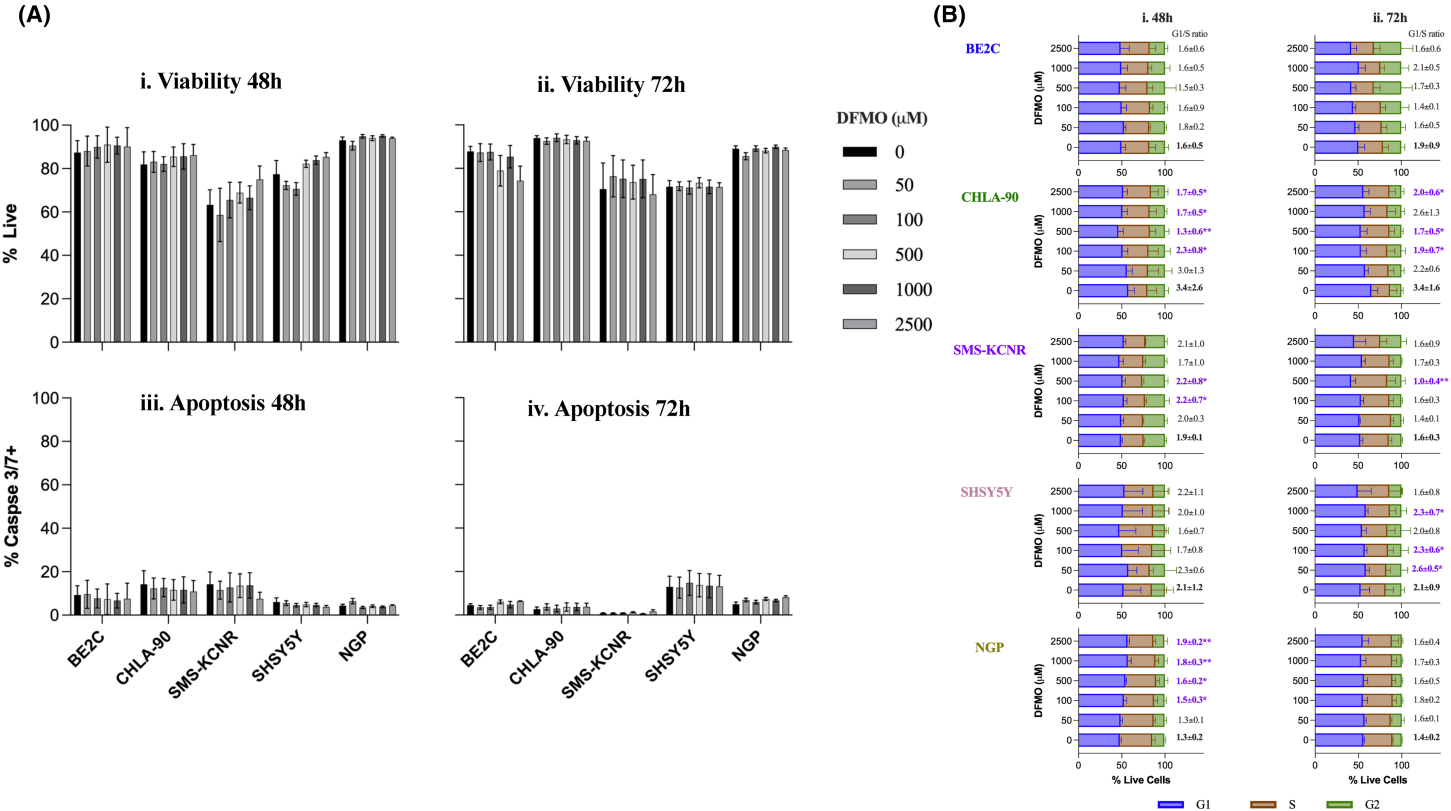
Effect of DFMO on cell viability, apoptosis, and cell cycle. Five established neuroblastoma cell lines (BE2C, CHLA‐90, SMS‐KCNR, SHSY5Y, and NGP) were treated with escalating doses of DFMO (0, 50, 100, 500, 1000, and 2500 μM) for 48 and 72 h. The cells were harvested and stained with a custom flow cytometry panel combining viability (fixable live/dead far red stain‐(Invitrogen), apoptosis markers (caspase‐3 and 7) (Vybrant FAM Casapse‐3 and 7 Assay Kit‐Invitrogen) and cell cycle stain (Vybrant DyeCycle™ Violet Stain‐Invitrogen). At least 100,000 live events were acquired on BD Fortessa cytometer and data analyzed using FlowJo software. Figure 1 (A) top panel shows median percentage of viable cells at each treatment for (i) 48 and (ii) 72 h, respectively, while the bottom panels (iii) and (iv) shows changes in apoptosis (*p* > 0.05). (B) shows median percentage of cells in G1, S and G2 cell cycle stages for (i)48 and (ii) 72 h post‐DFMO treatment.

We evaluated the effect of DFMO on NB cell lines for cell cycle, senescence, and cell viability after DFMO treatment by flow cytometry. We did not observe any dose‐dependent cell death nor reduction in viability in all cell lines tested at all DFMO doses at the two time points. This observation suggests that DFMO is non‐cytotoxic at the clinically physiological range of 50–200 μM. We then evaluated the effect of DFMO on induction of apoptosis by flow cytometry using caspase 3/7 readout. Similarly, we did not observe any significant change in the magnitude of apoptosis at 48 or 72 h timepoints across all the tested DFMO doses (*p* > 0.05) (Figures 1A and S2).

We next evaluated the effect of DFMO treatment on cell cycle. Although all cell lines responded at the physiologically relevant doses by undergoing cell cycle arrest, there were variations in magnitude of response across cell lines. Generally, BE2C and CHLA90 cell lines were more sensitive to DFMO cytostatic activity compared to SMS‐KCNR, SH‐SY5Y, and NGP at 100 μM for both 48‐ and 72‐h time points. The SMS‐KCNR cell line required a longer time and higher doses of DFMO treatment to exhibit cytostatic effect. After 48‐h and at 100 μM DFMO dosage, the most sensitive cell line was SMS‐KCNR (*p* = 0.0238) while the rest of the cell lines did not show any significant difference compared with the vehicle. However, at doses above 500 μM, all cell lines showed a reduction in proliferative ability. At 500 μM CHLA90 (*p* = 0.0317), and SMS‐KCNR (*p* = 0.0238) showed significant G1/S cell cycle arrest. NGP cell line, showed a cell cycle arrest trend at 500 μM (*p* = 0.0952) and this became significant at 1000 μM (*p* = 0.0494) and 2500 μM (*p* = 0.0238). Interestingly, CHLA90 cells lines seemed to reverse cell cycle arrest at higher DFMO concentrations of 1000 and 2500 μM. At 72‐h post‐treatment, CHLA90 show significant G1/S arrest at 500 μM (*p* = 0.0159) and 2500 μM (*p* = 0.0397) although the trend had been noted from 50 (*p* = 0.0635) and 100 μM (*p* = 0.0952) compared with vehicle. SMS‐KCNR showed a significant G1/S arrest at 500 μM (*p* = 0.0222) (Figure 1B).

### 3.2 DFMO treatment induces senescence‐associated β‐galactosidase activity while downregulating cyclin D1 and phosphor‐RB expression in neuroblastoma cell lines

**FIGURE 2:**
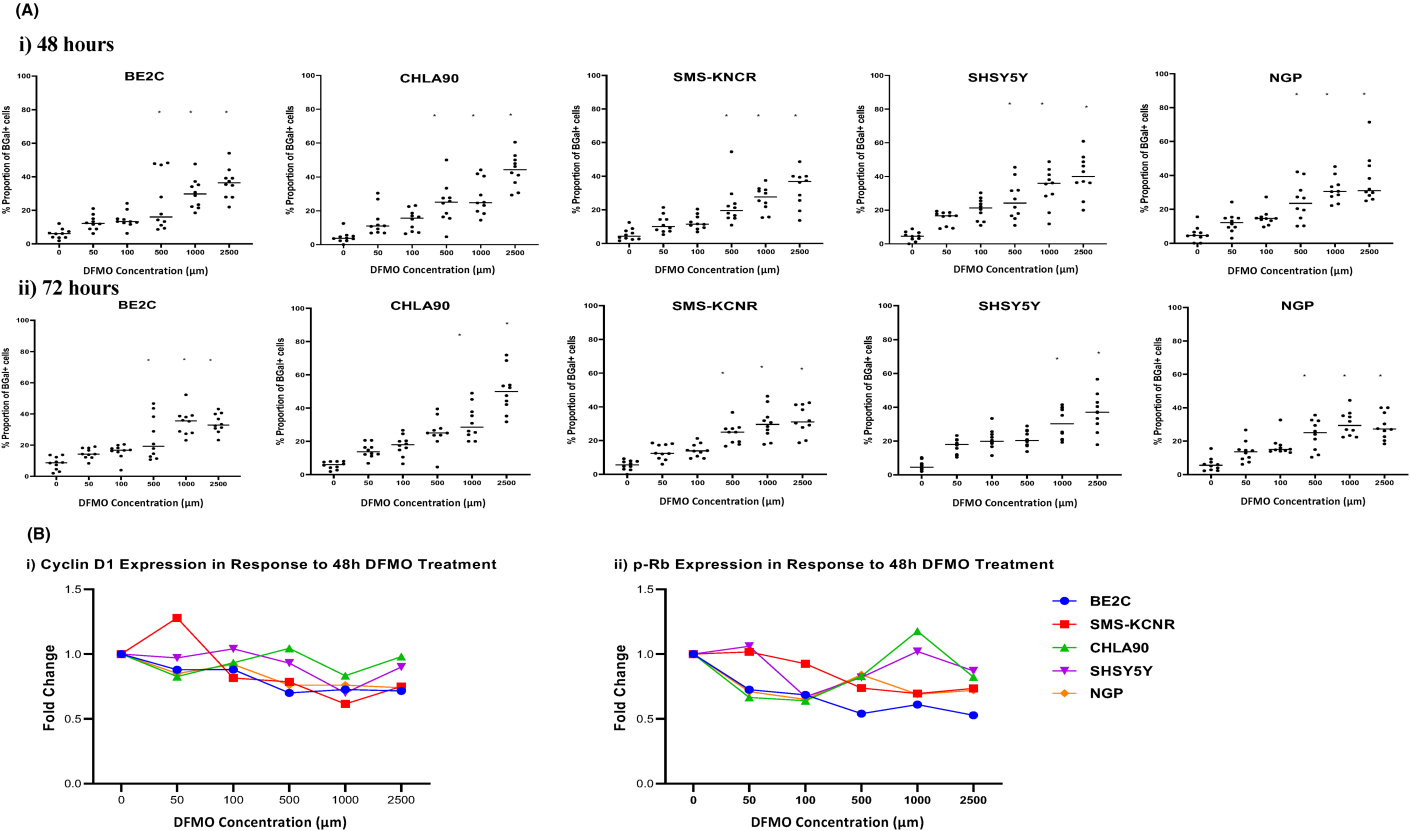
DFMO treatment increases senescence‐associated βGal while downregulating Cyclin D1 and phospho‐Rb expression in neuroblastoma cell lines. Neuroblastoma cells were seeded in 6‐well plates and treated with escalating doses of DFMO (0, 50, 100, 500, 1000, and 2500 μM) for 48 and 72 h. (A) The cells were fixed and stained for SA‐βGal activity following strict adherence to the manufacturers protocol. Positive cells were detected by the presence of blue color staining. Five different field of view images were taken at 20× magnification. The number of positive cells were counted and expressed as percentage of the total number of cells per field of view. We observed a dose‐dependent increase in SA‐βGal activity in cells treated with DFMO relative to untreated controls at both (i) 48 and (ii) 72 h. (B) Densitometry analysis of immunoblot staining for cell cycle‐associated proteins (i) cyclin D1 (CCND1) and (ii) phosphorylated Retinoblastoma protein (p‐Rb) demonstrated overall decreases in levels of both proteins at 48 h, a further indication of DFMO treatment initiating transition toward a senescent phenotype while inhibiting cell proliferation.

To investigate the effect on DFMO treatment on cell senescence, NB cell lines were treated with escalating DFMO doses of 0–2500 μM, and SA‐βGal activity was measured after 48 and 72 h. We observed increased SA‐βGal activity in DFMO‐treated wells compared to the untreated ones at both time points at the tested doses. Higher doses of DFMO induced SA‐βGal activity were comparable to that of positive control etoposide, which was a 6‐fold increase to that of the control (*p* > 0.0001) in all cell lines. We did not observe significant differences in SA‐βGal activity in 50 μM DFMO‐treated wells compared with controls in most the cell lines. We observed a dose‐dependent increase in SA‐βGal activity in BE2C cells across doses at both 48 (*p* > 0.0001) and 72 h (*p* = 0.0118). There was no significant difference in activity at lower dose (50 μM) in BE2C cells (*p* < 0.005) at 48 h or at 72 h (*p* = 0.001) (Figure 2A(i) and (ii)).

To investigate the effect of DFMO treatment on transcription and translation of proteins associated with tumorigenesis, the protein levels of Phosphor‐RB and Cyclin D1 was measured in cell lysates from the above treatments in all cell lines at 48 h. Although we did not observe a significant difference between doses, there was an overall trend of down regulation of both Phosphor‐RB and Cyclin D1 across cell lines suggesting an initiation of senescence phenotype while inhibiting cell proliferation (Figure 2B).

### 3.3 DFMO inhibits neurosphere formation and progression in neuroblastoma cell lines

We investigated the effect of DFMO on sphere initiation, formation, and development in different cell lines. We tested our hypothesis that DFMO interferes with neurosphere formation using 5 established human NB cell lines with *MYCN* amplification (BE2C, SMS‐KCNR, and NGP cell lines) or without (CHLA90, and SH‐SY5Y cell lines). We evaluated the effect of 0–2500 μM DFMO over 4 weeks measuring size and frequency of neurospheres. We observed that dose and time influenced changes in the number and size of tumor spheres.

**FIGURE 3:**
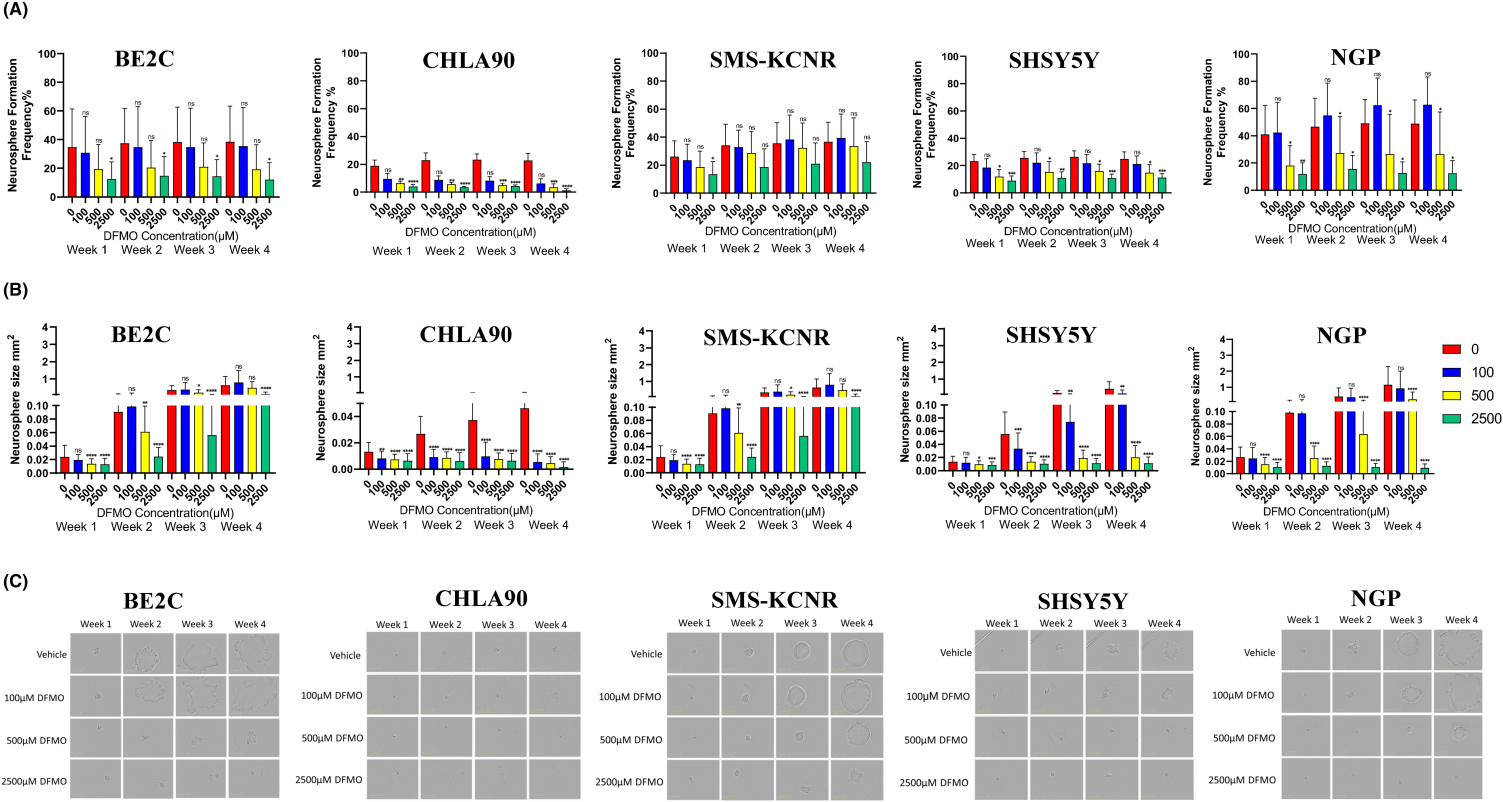
Increasing doses of DFMO abrogates neurosphere formation and progression in neuroblastoma cell lines. Two cells per well for each cell line were seeded in 96‐well plates and treated with DFMO doses of 0, 100, 500, and 2500 μM. The frequency and the size of neurospheres were monitored and imaged weekly using Incucyte® ZOOM for 4 weeks. The top panel (A) is graphical representation of neurosphere numbers over time while the lower panel (B) shows the changes in neurosphere sizes over time in the tested cell lines. The bottom panel (C) shows representative images of neurosphere over time. We observed a dose‐dependent inhibition on the frequency and size of neurospheres in the five cell lines tested over time. Significant changes were observed in four of the cell lines (CHLA90, SHSY5Y, BE2C, and NGP) at 500 μM and 2500 μM compared to the vehicle.

Here, we show that DFMO either slows down or abrogates neurosphere formation in all the five cell lines at the tested conditions (*p* > 0.001). We observed differences in frequencies and sizes of neurospheres between cells lines over time. The increase in neurosphere frequencies and size in vehicle control was curtailed by space limitation. We observed large neurospheres in BE2C, SMS‐KCNR, and NGP cell lines while CHLA90 and SHSY5Y had the smallest and least numbers of neurospheres over time. The BE2C cell line formed large neurospheres and grew faster than SMS‐KCNR, although both cell lines are *MYCN* amplified. The SMS‐KCNR cell line formed numerus neurospheres albeit of smaller sizes. The CHLA90 and SHSY5Y which do not contain *MYCN* amplification were most sensitive to DFMO treatment. There was near complete abrogation of neurosphere initiation and progression in culture wells that contained DFMO concentration of 2500 μM compared with the vehicle (*p* = 0002) after the first week of treatment. The greatest drop in neurosphere formation was in CHLA90 cells (80%–90% drop), (*p* = 0.0164); while NGP and SHS5Y5 retained about 1/3 neurosphere formation (*p* = 0.0073 and *p* = 0.002 respectively) and BE2C had 50% retention compared to the control (*p* = 0.04). Taken together, our data suggest that DFMO doses at the physiological limits can induce these effects. Interestingly, we observed that cells with *MYCN* amplification required slightly higher doses of DFMO to exert the same effect as compared to those without (Figure 3A–C). Our data thus demonstrate that DFMO decreases the tumor‐initiating potential and progression in vitro.

### 3.4 DFMO inhibits tumor initiation, progression and growth in mouse models

#### 3.4.1 Pretreatment of NB cells with DFMO inhibits neurosphere formation in vitro and tumor initiation/formation in vivo

We demonstrated cell cycle arrest, induction of senescence and abrogation of neurosphere formation with DFMO treatment in vitro. Next, we explored the effect of DFMO pretreatment on tumor formation in vitro and in xenograft models. BE2C and SMS‐KCNR cells were cultured in the presence or absence of DFMO (5 mM) for 10 and 20 days. Remaining viable cells were then plated in untreated media and assessed for neurosphere formation. Pretreatment with DFMO for 10‐ and 20‐day inhibited neurosphere formation in both cell lines (20‐day pretreatment *p* = 0.012 for BE2C and 20‐day pretreatment *p* = 0.014 for SMS‐KCNR).

**FIGURE 4:**
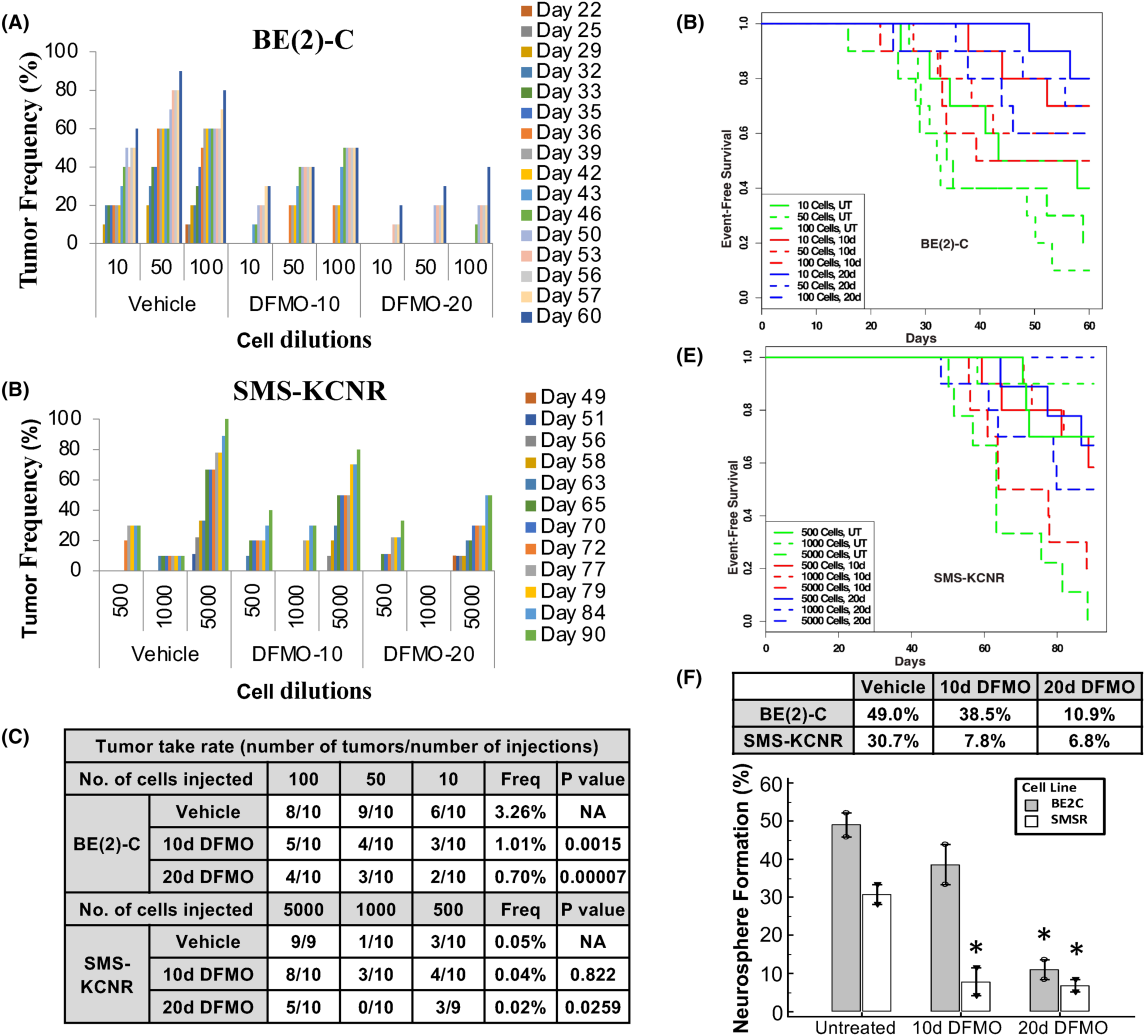
Effect of in vitro DFMO pretreatment on tumor formation/initiation capacity of neuroblastoma cells. (A) Xenograft tumor model for limiting dilution analysis (LDA) using BE2C cells: mice were injected with 10, 50, or 100 cells pretreated for 10 days or 20 days with DFMO (5 mM) and tumor frequency was evaluated for 60 days. (B) The number of mice in each Xenograft group for LDA using BE2C and SMS‐KCNR cells. Mice were injected with 500, 1000, or 5000 cells pretreated for 10 or 20 days with DFMO (5 mM) and tumor frequency was evaluated for 90 days. (C) LDA of tumor takes at study termination. Tumor‐initiating cell frequencies and *p*‐values are calculated using online ELDA platform (accessed 20 September 2016), followed by post hoc log‐rank and Fisher's test analysis. Overall *p*‐value, *p* < 0.4. (D) Event‐free survival plots of xenograft mice injected with BE2C cells pretreated with DFMO (5 mM) for 0, 10, or 20 days. ANOVA *p*‐value evaluating the significance of DFMO effect for length of treatment, *p* < 0.001 compared to vehicle control. (E) Event‐free survival plots of xenograft mice injected with SMS‐KCNR cells pretreated with DFMO (5 mM) for 0, 10, or 20 days. ANOVA *p*‐value, *p* < 0.05 compared to vehicle control. (F) Neurosphere assay reporting tumor formation frequencies for DFMO‐pretreated NB cells. BE2C and SMS‐KCNR cells were pretreated with DFMO for 0, 10, or 20 days and allowed to form neurospheres in neurobasal media for 2 weeks.

Leveraging on extreme limiting dilution analysis (ELDA) assay, (used to measure the frequency of tumor‐initiating cells within a tumor cell population), we further investigated the effect of DFMO on tumor associated functional end points using xenograft mouse models with DFMO‐pretreated NB cells. Limiting dilutions of either control or pretreated viable BE2C and SMS‐KCNR cells (either 10 day or 20‐day pretreatment) were injected subcutaneously into right flanks of nude mice (10 mice per group). Mice were subsequently followed for tumor formation for 60 and 90 days, respectively. Tumor formation was significantly decreased in xenograft mice injected with BE2C or SMS‐KCNR cells pretreated with DFMO at 5 mM DFMO (BE2C, *p* < 0.001; SMS‐KCNR, *p* < 0.05) (Figure 4A,B). We observed increased suppression of tumor‐initiating capacity with increase in the number of days of DFMO pretreatment. Although 10 days of DFMO pretreatment significantly decreased the frequency of tumor‐initiating cells for BE2C (*p* < 0.0015), there was more pronounced suppression of tumor formation following 20 days of pretreatment in both BE2C and SMS‐KCNR cells (*p* < 0.0001 and *p* < 0.05, respectively) (Figure 4C). BE2C and SMS‐KCNR event‐free survival plots (Figure 4D,E, respectively) further illustrate a suppressive effect of DFMO on tumor‐initiating cells, showing improvement in time‐to‐event survival (BE2C *p* < 0.001, SMS‐KCNR *p* < 0.05). Together, these data demonstrate that DFMO pretreatment abrogates tumor initiation in BE2C and SMS‐KCNR cells preventing tumor formation in xenografts.

#### 3.4.2 DFMO treatment inhibits tumor initiation/formation in limiting dilution xenograft mouse models

**FIGURE 5:**
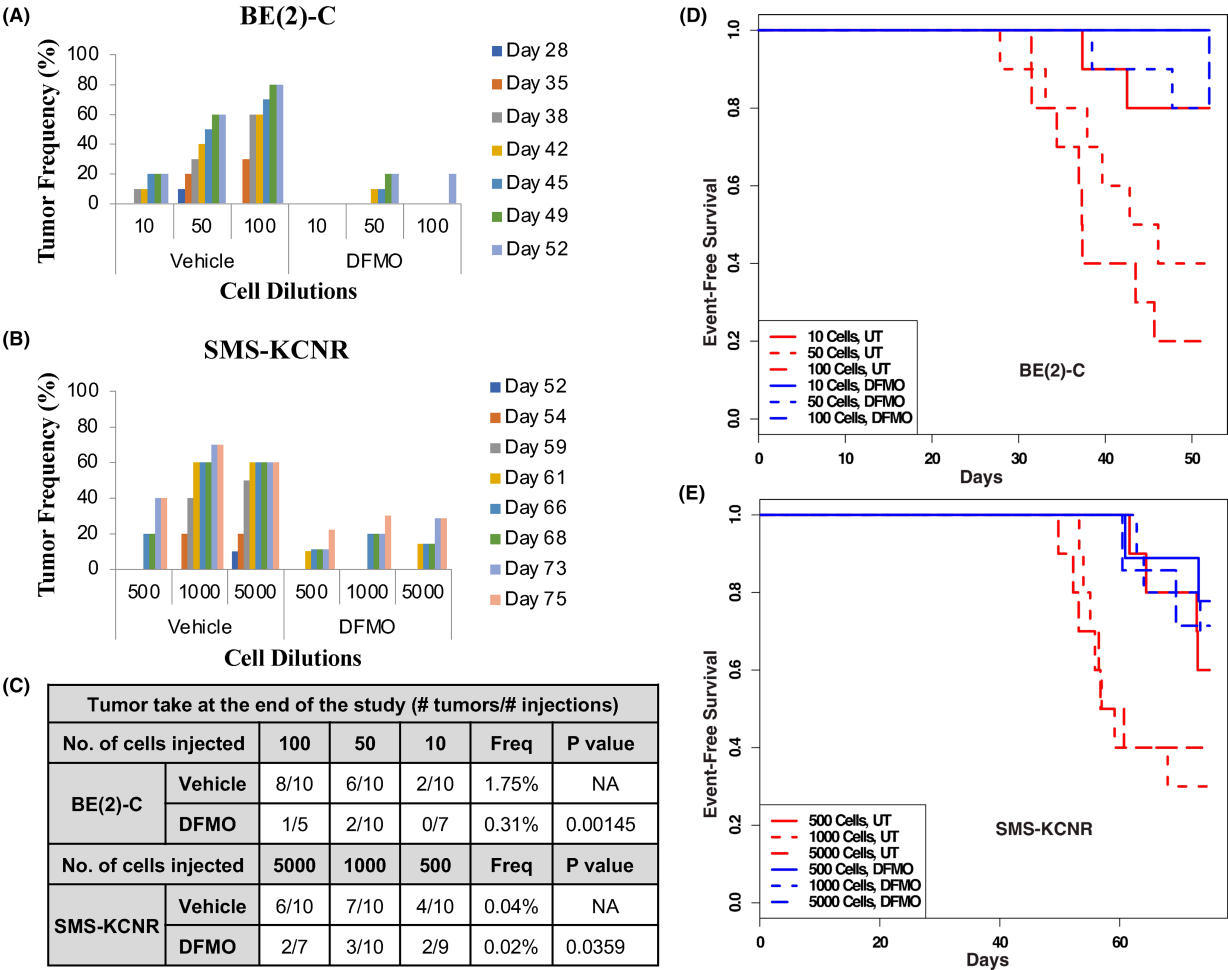
Effect of in vivo DFMO treatment on tumor formation/initiation capacity of NB cells. (A) Xenograft for LDA using BE2C cells. Mice were injected with 10, 50, or 100 cells and treated with DFMO (2%) in drinking water. Tumor frequency was evaluated for 52 days. (B) Xenograft for LDA using SMS‐KCNR cells. Mice were injected with 500, 1000 or 5000 cells and treated with DFMO (2%) in drinking water. Tumor frequency was evaluated for 75 days. (C) Frequencies, and *p*‐values are calculated using online ELDA platform followed by post hoc log‐rank and Fisher's test analysis. Overall *p*‐value, *p* < 0.04 compared with vehicle control. (D) Event‐free survival plots of xenograft mice injected with BE2C cells and treated with DFMO (2%) in drinking water. Overall *p*‐value, *p* < 0.001 compared to vehicle control. (E) Event‐free survival plots of xenograft mice injected with SMS‐KCNR cells and treated with DFMO (2%) *in* drinking water. Overall *p*‐value, *p* < 0.005 compared to vehicle control. The number of mice in each group are depicted in 5C.

DFMO‐pretreated BE2C and SMS‐KCNR cells displayed a significant suppression in tumor initiation and formation in pretreated ELDA xenografts (*p* = 0.031). As shown above, pretreatment of cells with DFMO for 10 days inhibited neurosphere formation in BE2C (*p* = 0.012) and SMS‐KCNR (*p* = 0.014). To assess the ability of in vivo DFMO treatment to reduce tumor initiation and formation, we employed an additional ELDA xenograft mouse model. Nude mice were injected with limiting dilutions of BE2C cells or SMS‐KCNR cells. The mice then either received untreated drinking water or water supplemented with 2% DFMO. Tumor formation was observed with increasing cell concentrations in untreated mice from 20% to 80% for BE2C and 40%–70% with SMS‐KCNR cells (*p* = 002) (Figure 5A). Conversely, mice receiving DFMO‐supplemented water had significantly lower rates of tumor formation as determined by ELDA (BE2C, *p* < 0.001; SMS‐KCNR, *p* < 0.05) (Figure 5A–C). Mice receiving DFMO in drinking water also displayed delayed tumor formation and increased event‐free survival rate, as compared to control group (BE2C, *p* < 0.001; SMS‐KCNR, *p* < 0.005) (Figure 5D–F). Together, these data demonstrate that DFMO pretreatment is sufficient to decrease the frequency of tumor‐initiating cells within BE2C and SMS‐KCNR NB cells and increased event‐free survival.

### 3.5 DFMO inhibits tumor growth/progression, neurosphere formation and oncoprotein expression in ex vivo xenograft mouse models

**FIGURE 6:**
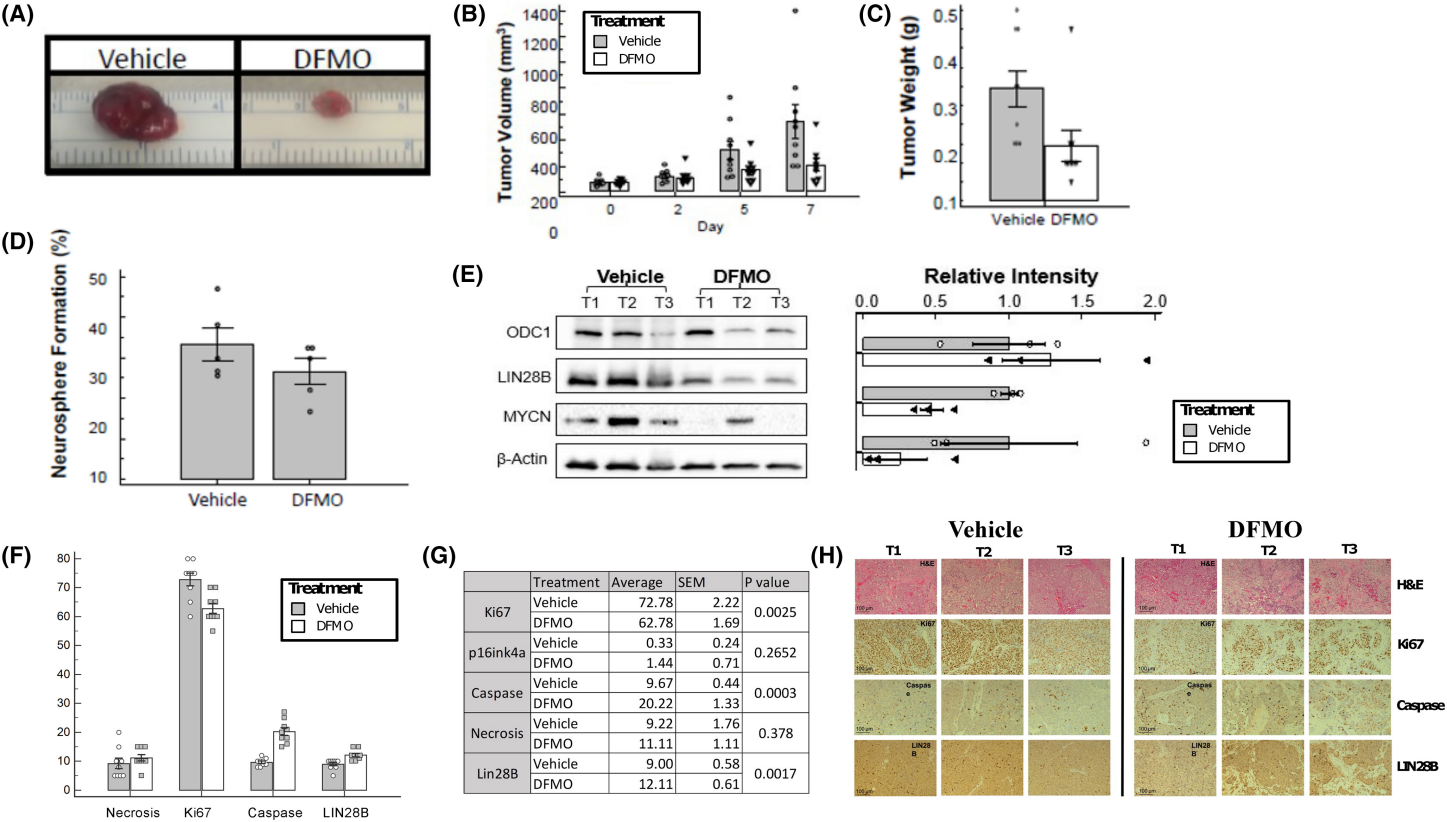
Effect of DFMO treatment on tumor growth and progression in mice. Mice were injected with 2 × 10^6^ BE2C cells. After tumors formed (7 days), the mice were given either normal drinking water (vehicle) or DFMO (2%)‐containing water. Following 7 days of treatment, the tumors were (A) resected and measured for size (B) volumes and (C) weights. The values are expressed as means ± *SE* (*N* = 3). 2‐way ANOVA, *p* < 0.001 compared with vehicle control. (D) Tumor cells dissociated from vehicle‐treated or DFMO‐treated mice were evaluated for neurosphere formation frequency for 2 weeks. The values are expressed as means ± *SE* (*N* = 5, 96‐well plates). 1‐way ANOVA followed by Fisher's *t*‐test, *p* < 0.005 compared with vehicle control. (E) Dissociated tumor cells were assayed for protein expression ODC1, LIN28B and MYCN by Western blot analysis. Band intensities were quantified and normalized to β‐actin (*n* = 9). Western blot images are depicted as 3 representative tumors from each group (T1–T3). (F) Bar graph depicting the percentage of xenograft tissue positive for each endpoint. (G) Tabular representation of IHC scoring, including the average score, standard error of the mean, and *p*‐value for each group; necrosis cellular senescence (p16ink4a), proliferation index (Ki67), apoptosis (cleaved caspase 3), and LIN28B (*N* = 9 for each group). (H) Representative immunohistochemistry of three tumors from each group (T1–T3) were stained for tumor structure by hematoxylin and eosin (H&E), proliferation index (Ki67), apoptosis (cleaved caspase 3), and LIN28 expression. *N* = 9 for the number of tissue slices quantified for each end.

Given that in vitro treatment of NB cells with DFMO resulted in decreased neurosphere formation, we sought to determine whether these endpoints could be recapitulated with cells from tumors excised from DFMO‐treated mice. We also sought to confirm whether DFMO treatment affected *MYCN* and LIN28 expression. Therefore, mice were injected with BE2C cells and allowed to form tumors, after which point mice received either untreated water or water supplemented with 2% DFMO for 7 days. After 7 days, tumors were resected, and the volume measured (Figure 6 A–C). Tumors were then dissociated, and the resultant cells either plated for neurosphere formation assays or lysed for western blot analysis. Tumors from DFMO‐treated mice displayed a significantly lower tumor volume (Figure 6B, *p* < 0.001) and tumor weight (Figure 6C, *p* = 0.027) relative to vehicle control. The NB cells dissociated from mouse tumors treated with DFMO trended toward a reduction in neurosphere formation (Figure 6D). DFMO treatment trended toward reduction in protein expression for LIN28B, and *MYCN* compared to vehicle, suggesting a simultaneous ex vivo reduction in our previously described endpoints,^17^ following in vivo administration of DFMO (Figure 6E,F). The tumor volume data demonstrates a pronounced inhibition of cell growth with in vivo treatment of DFMO (*p* = 0.003). We therefore utilized immunohistochemistry to analyze tumor structure (H&E), cell division (Ki67), cellular senescence (p16ink4a), cell death (cleaved caspase 3), as well as LIN28B (Figure 6G,H). Our data show increased cell death with concomitant induction of apoptosis, as evidenced by increased levels of cleaved caspase 3 (*p* = 0.0003). In addition to induction of caspase, the data also demonstrate reduced Ki67 levels (*p* = 0.002), which is indicative of a loss proliferative state as well as a non‐significant increase in necrosis and senescence (p16ink4a). Moreover, DFMO treatment slightly increased the proportion of cells that stained positive for LIN28B (*p* = 0.002).

## 4 DISCUSSION

Tumor relapse after completion of standard treatment contributes significantly to childhood death due to cancer.^22^ Understanding the mechanisms that herald tumorigenesis is important to the development of novel antineoplastic agents. Preventing relapse through maintenance therapy is one approach that has proven successfully in many cancers, most notably in pediatric leukemia where the survival rates are now over 90% with extensive maintenance therapy.^23^ This concept is under evaluation in many studies, including the recent published clinical trials of DFMO as a maintenance therapy in NB, with increased survival rates and well‐tolerated adverse effects.^24^, ^25^ These promising results from our group^25^ led us to hypothesize that DFMO interferes with cellular processes that are integral to tumorigenesis.

Tumor cells exhibit altered cellular processes in terms of proliferation, resistance to apoptosis, increased invasiveness, and metastatic activity.^26^ Experimentally, tumor‐associated functional endpoints have been used as functional readouts to study the effects of drugs on these processes.^27^ These endpoints include measurement of tumor associated protein expression as well as the ability of cells to form tumor spheres in culture, both of which can be indicative of cellular potential towards tumor initiation and progression.^26^, ^28^, ^29^, ^30^, ^31^ We believe that the clinical benefit of DFMO could be due to induction of permanent loss of tumor‐initiating ability. Although we did not observe significant changes in cell numbers, or viability, we noted cell cycle arrest across all five cell lines tested. We observed that some cells required higher doses of DFMO or longer treatment periods of time for a measurable effect. There was no observable change in cell death due to DFMO dosages over time suggesting that DFMO is not cytotoxic but cytostatic. Different drugs have different pharmacodynamics and kinetics in individuals which may be influenced by culture dynamics.^32^

Neoplastic agents induce senescence via genotoxic stress, hyperactivation of mitotic signaling or oxidative stress leading to stable cell cycle arrest.^33^, ^34^ This DNA hyper damage is accompanied by increased lysosomal enzyme SA‐βGal activity. SA‐βGal activity is routinely used as a functional readout of senescence in vitro. The induction of senescence has proven beneficial in suppressing tumor growth, progression, and metastasis.^33^, ^34^, ^35^ We observed increased SA‐βGal activity with DFMO treatment in all cell lines at the tested doses.^35^ This state, where cells switch to a stable non‐proliferative but metabolically active state in response to exogenous or endogenous stress, could be a mechanism through which DFMO inhibits tumorigenesis in neuroblastoma. Although cells undergoing senescence evade prolonged genetic stress and cumulative DNA damage,^36^ stable therapeutic DFMO levels in the blood stream through maintenance dosing permanently impedes their capacity to proliferate or form neurospheres, interfering with the cell physiology associated with tumorigenesis.^24^ Although we observed some differences in the degree of SA‐βGal activity between cells lines, there was an overall induction of senescence in all cell lines tested in a dose‐dependent manner. Previously through a phase 1 trial in patients with relapsed/refractory neuroblastoma, we demonstrated that DFMO concentrations of 9.54 μm/mL (52.24 μM) or 500 mg/m^2^ to 30.71 μg/mL (168.10 μM) or 1500 mg/m^2^ was well tolerated.^24^ In this study, the highest concentration of DFMO in the blood was measured to be slightly greater than 150 μM, suggesting that the doses required to target the NB cells are clinically achievable doses.^24^ The induced senescent cells have been shown to lose proliferative ability even after halting DFMO treatment.^37^ It would be interesting to investigate the longevity of these cells in circulation to identify if they have putative markers that can be targeted for immune clearance. Nonetheless, our observation makes DFMO an attractive compound for long‐term management of malignant tumors that have high relapse rates.

Sphere forming cells retain totipotent ability and exhibit greatest propensity to form cellular aggregates that recapitulate tumor formation in vitro.^38^ Neurosphere formation and development (increase in size and number) is an excellent functional readout for tumor initiation, progression, and development.^38^, ^39^ The ability to initiate neurosphere formation and subsequent progression has been correlated with poor prognosis in patients with solid tumors.^39^ A compound that vitiates neurosphere progression potentially offers promise in cancer management. We hypothesized that the success of DFMO as a maintenance drug in NB patients in remission^18^ was through abrogation of new tumor initiation. Previous studies that have attempted to characterize DFMO effects in vitro have utilized doses beyond the physiologically and clinically relevant limits. Furthermore, this has been described in only a limited number of cell lines and for a limited period of time.^17^ In our study investigating 5 cell lines over 4 weeks of DFMO treatment, all cell lines showed inhibition of neurosphere formation compared to the vehicle control. Initially, we observed neurosphere formation in treatment wells, albeit in small numbers and sizes compared with the control wells. Indeed, previous studies on antineoplastic drugs have shown that most cytostatic agents exert their effect slowly over a narrow therapeutic window. Prior to observable changes, DFMO could be initiating or altering the cellular processes senescence or apoptosis that are integral to tumorigenesis. During the assay, we observed morphological changes in the shape and outline of neurospheres between the vehicle and treatment groups. Neurospheres that formed in treatment wells had uneven outlines suggesting that DFMO interferes with plasma membrane integrity as well, though this has not been tested. Future studies will explore the effect of DFMO treatment on cell membrane integrity as another possible mechanism of its action. However, we noticed at 100 μM DFMO, in some cell lines like BE2C and SMS‐KCNR the size and frequency of neurospheres were comparable to that of the vehicle control. It can be noted that at this concentration, which is the clinically and physiologically relevant dosage, the cells may be exploiting alternative metabolic pathway in polyamine synthesis due to DFMO inhibition. Cancer cells normally develop subversive pathways to supply energy and structural blocks for cell division. This mechanism is actively under study in our laboratory.

The process of tumorigenesis is associated with altered gene expression and uncontrolled metabolic processes that do not conform to the normal cellular process. In various NB initiation and progression models, elevated levels of LIN28B, *MYCN* and ODC1 have been associated with increased tumor initiation and progression due to increased proto‐oncogene activity and ensuing progenitor‐like cells.^20^, ^40^, ^41^, ^42^ Conversely, the cleavage of caspase 3 and Rb proteins have been associated with senescence and cell death as they are suppressor proteins.^33^, ^43^, ^44^ Previous studies demonstrate that DFMO treatment extends tumor latency and survival in NB‐prone mice that overexpress *MYCN* in neural crest cells.^45^ Our data show that DFMO treatment leads to decreased levels of ODC1, Cyclin D1 and phospho‐Rb expression. ODC1 blockade has previously been shown to deplete levels of polyamines,^45^ while polyamine depletion has been directly correlated with a decrease in Cyclin D1 levels, and subsequently a decrease in the phosphorylation of Rb, resulting in G1 cell cycle arrest.^10^, ^46^, ^47^, ^48^ At the same time, DFMO decreases LIN28B and *MYCN* protein levels, both of which are associated with driving cellular proliferation. By pleiotropically targeting multiple cellular proliferation foci, DFMO inhibits cell division and induces apparently irreversible cellular senescence.

Our xenograft studies further confirmed these results, showing that tumor formation was significantly delayed, and event‐free survival rate was increased in xenograft mice following DFMO pretreatment of NB cells. Additionally, our present studies implemented two ELDA xenograft models as a measure of tumor‐initiating cell frequency in the tumor cell population. The first model utilized DFMO‐pretreated cells, while the second model was performed in DFMO‐treated mice. Each confirmed a decrease in the tumor‐initiating cell frequency of the tumor populations when treated with DFMO preventing tumor formation. Furthermore, these results suggest that there may be a permanent decrease or loss of tumor‐initiating BE2C and SMS‐KCNR cells and that the results of in vitro treatment are reproducible via in vivo treatment models. These data provide evidence that DFMO may increase survival outcome for high‐risk patients through selective inhibition or depletion of tumor‐initiating cells.

In addition to the inhibitory effects of DFMO on tumorigenesis, our IHC data demonstrate that DFMO induces cell death, both through decreased proliferation and through increased apoptotic signaling pathways in mice bearing BE2C and SMS‐KCNR xenografts. This was unexpected as in vitro treatment with DFMO demonstrates that the drug largely acts in a cytostatic manner. Although reduced proliferation index was observed in DFMO‐treated mice, these mice also displayed a significant induction of caspase activity. This may be due to several biological factors that in vitro studies cannot recapitulate, such as effects upon angiogenesis and synergy with immune cells. Interestingly, the expression of p16ink4a, a common tumor suppressor frequently used to identify senescent cells, trended toward an increase in expression in mice that received DFMO in drinking water. The average expression level, however, was still relatively low (slightly above 1%). These data may not be discordant with our previous in vitro results, however, as our flow cytometry data suggest that physiological levels of DFMO preferentially induce expression of senescence correlated marker. It is also plausible that 1‐week treatment was not sufficient to induce significant increase in p16ink4a. There is need for further studies to determine the mechanism by which DFMO induces cell death within tumor cells.

Further studies are underway to define the mechanisms of DFMO in NB tumorigenesis and determine if its effects are permanent or transient. Our data imply that DFMO selectively targets tumor cell population preventing relapse in high‐risk neuroblastoma patients. Furthermore, DFMO appears to be pleiotropic in its anti‐NB activity as we observe multiple cumulative effects on cellular processes leading to growth inhibition over time even for the least sensitive cell lines.

## AUTHOR CONTRIBUTIONS

**Divya Gandra:** Investigation (equal); methodology (equal); writing – review and editing (equal). **David H. Mulama:** Data curation (equal); formal analysis (equal); investigation (equal); writing – original draft (equal). **David M. Foureau:** Data curation (equal); formal analysis (equal); investigation (equal); methodology (equal); project administration (equal); supervision (equal); writing – review and editing (equal). **Kimberly Q. McKinney:** Data curation (equal); investigation (equal); methodology (equal); writing – review and editing (equal). **Elizabeth Kim:** Data curation (equal); investigation (equal); writing – review and editing (equal). **Kaitlyn Smith:** Data curation (equal); formal analysis (equal); investigation (equal); methodology (equal); project administration (equal); writing – review and editing (equal). **Jason Haw:** Data curation (equal); investigation (equal); methodology (equal); writing – review and editing (equal). **Abhinav Nagulapally:** Formal analysis (equal); methodology (equal); writing – review and editing (equal). **Giselle L. Saulnier Sholler:** Conceptualization (lead); data curation (equal); formal analysis (equal); funding acquisition (equal); methodology (equal); project administration (equal); supervision (lead); writing – review and editing (lead).

## FUNDING INFORMATION

This work was supported by the Beat Childhood Cancer Foundation, Brooke's Blossoming Hope Foundation, Ethan's Rodeo, Meryl, and Charles Witmer Foundation.

## CONFLICT OF INTEREST STATEMENT

All data included in this manuscript are freely available by contacting the corresponding author. All authors declare no potential conflict of interest.

## ETHICS STATEMENT

All animal experiments were conducted in accordance with the institutional animal care and use protocol (IACUC) approved by the West Michigan Regional Laboratory (WMRL) as per the Committee on Animal Research and Ethics (CARE) guidelines.

## Supporting information


Figure S1.

Figure S2.

Figure S3.

Data S1.

## Data Availability

The data used in this publication are freely available by contacting the corresponding author.
