# Supplementary figures and images for: DFMO inhibition of neuroblastoma tumorigenesis
Source: Cancer Med. 2024 Apr 30;13(9):e7207. doi: 10.1002/cam4.7207 (PMC11058673; doi:10.1002/cam4.7207)

# Supplementary Figure 1: Animal Studies Schema

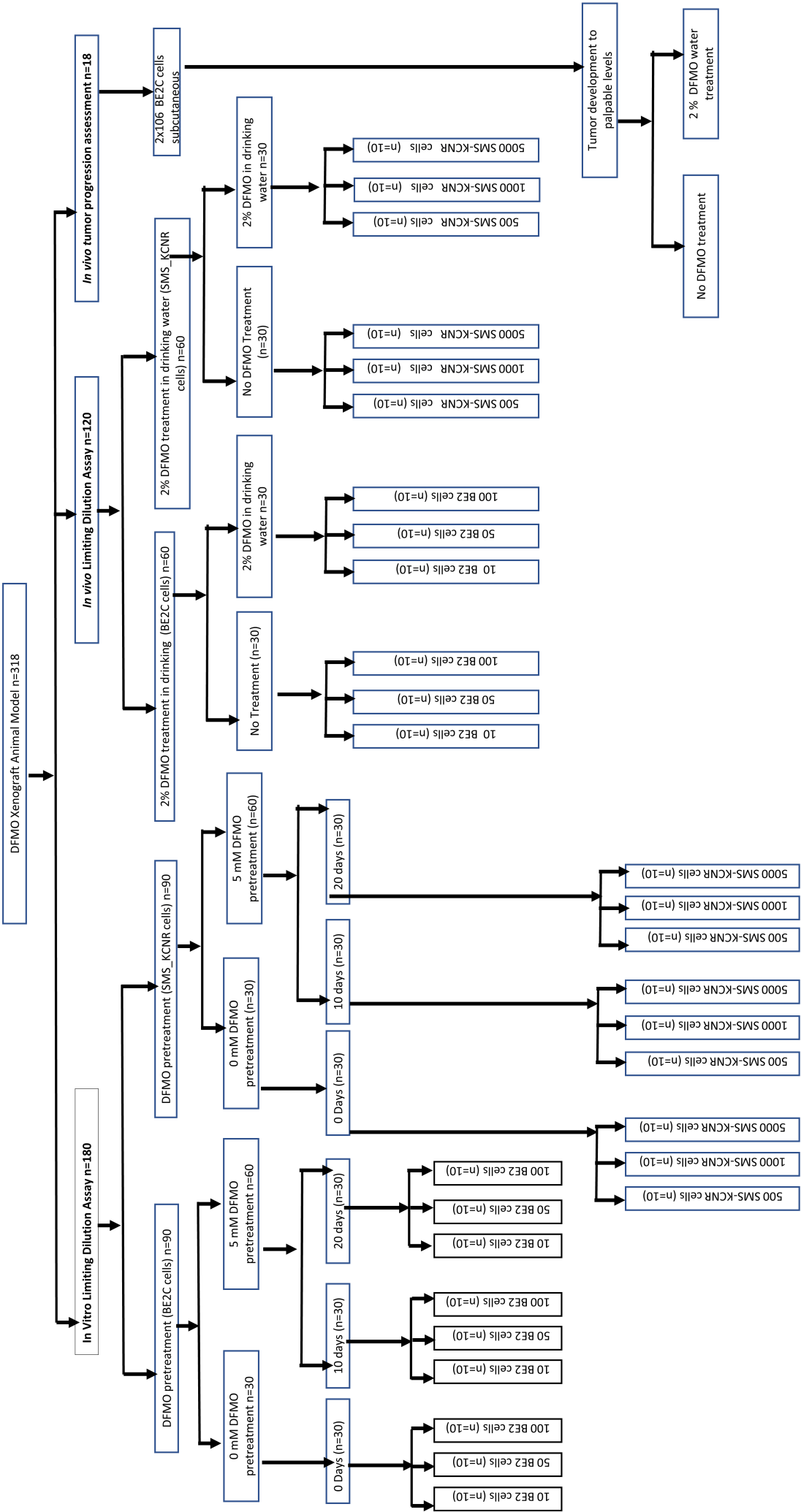

Supplement: Supplementary file 1 — Figure S1. [file CAM4-13-e7207-s003.pdf]
