# Supplementary material for: DFMO inhibition of neuroblastoma tumorigenesis
Source: Cancer Med. 2024 Apr 30;13(9):e7207. doi: 10.1002/cam4.7207 (PMC11058673; doi:10.1002/cam4.7207)

Supplementary Figure 2: Escalating doses of DFMO induces cell cycle arrest in neuroblastoma cell lines

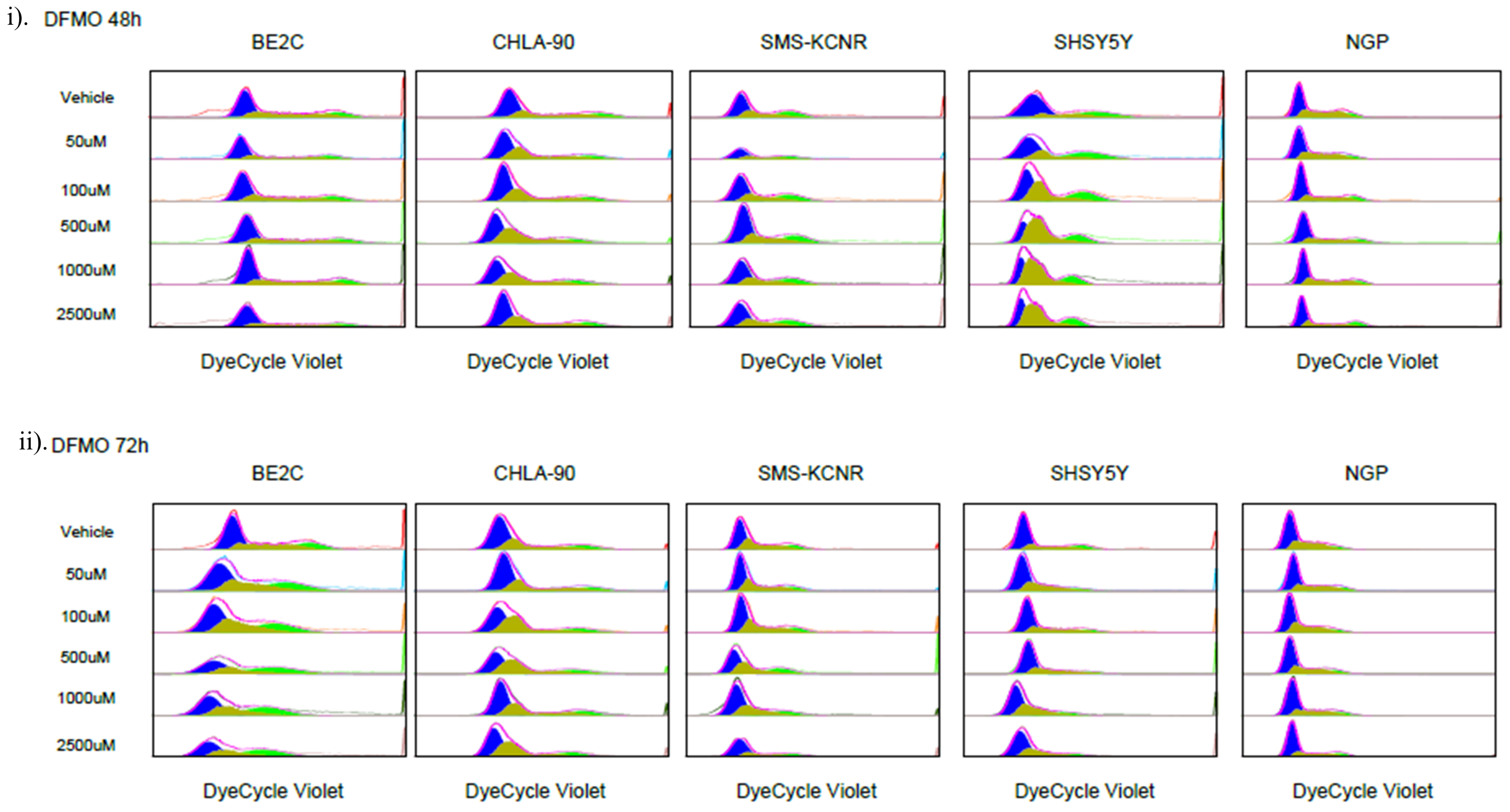

Supplement: Supplementary file 2 — Figure S2. [file CAM4-13-e7207-s004.pdf]
