# Supplementary material for: DFMO inhibition of neuroblastoma tumorigenesis
Source: Cancer Med. 2024 Apr 30;13(9):e7207. doi: 10.1002/cam4.7207 (PMC11058673; doi:10.1002/cam4.7207)
Supplement: Supplementary file 3 — Figure S3. [file CAM4-13-e7207-s001.pdf]

Supplementary Figure 3: Images of Tumors following *In vivo* DFMO Treatment in Animal Model Experiments

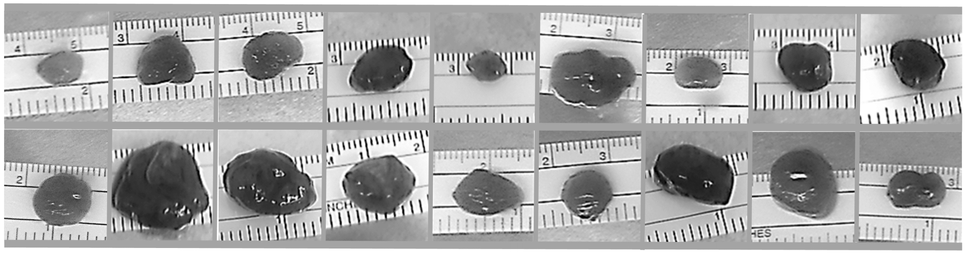

Treated

Untreated
