# Supplementary material for: DFMO inhibition of neuroblastoma tumorigenesis
Source: Cancer Med. 2024 Apr 30;13(9):e7207. doi: 10.1002/cam4.7207 (PMC11058673; doi:10.1002/cam4.7207)
Supplement: Supplementary file 4 — Data S1. [file CAM4-13-e7207-s002.docx]

**FIGURE LEGENDS -Supplementary Figures**

**Supplementary Figure 1. Animal Studies Schema**

4-week-old female nude (nu/nu) mice were used for this study. Using Limiting Dilution Assay both *in vitro* and the mice were divided into 3 study arms. The first arm investigated the inhibitory effect of DFMO on *in-vitro* NB pretreated (BE2C and SMS-KCNR) cells which were subcutaneously injected in mice and the ability of the cells to initiate and form tumors monitored over time. The second arm explored the effect of DFMO treatment on preformed tumors. Mice were subcutaneously injected with NB cells (BE2C and SMS-KCNR) and then treated with 2% DFMO in drinking water. The third arm involved injecting BE2C cells in mice, allowing tumors to form and then treating the mice with DFMO in drinking water. Tumor initiation and progression was monitored throughout the study. Excised tumors were measured for size and weight and then dissociated for western blot assay. We observed a reduction in tumor size, weight and volume with DFMO treatment group compared to the control group.

**Supplementary Figure 2: Histograms showing the Effect of DFMO treatment of cell cycle**

NB cell lines (BE2C, CHLA-90, SMS-KCNR, SHSY5Y and NGP) were treated with varying concentrations of DFMO (0, 50, 100, 500, 1000 and 2500µM) for 48 and 72 hours. Presented are histograms showing cell cycle arrest in different cell lines at i) 48 and ii) 72h post treatment.

**Supplementary Figure 3: Images of Tumors Following in vivo DFMO Treatment in Animal Model Experiments**

Mice were injected with 2x10^6^ BE2C cells. After tumors formed (7 days), the mice were given either normal drinking water (vehicle) or DFMO (2%)-containing water. Following 7 days of treatment, the tumors were resected and measured for volumes and weights. Mice treated with DFMO has relatively smaller tumors compared to the controls showing *in vivo* DFMO treatment inhibits progression of tumors in BE2C xenograft mice.
